# A risk-based subgroup analysis of the effect of adjuvant S-1 in estrogen receptor-positive, HER2-negative early breast cancer

**DOI:** 10.1007/s10549-023-07099-4

**Published:** 2023-09-07

**Authors:** Masahiro Takada, Shigeru Imoto, Takanori Ishida, Yoshinori Ito, Hiroji Iwata, Norikazu Masuda, Hirofumi Mukai, Shigehira Saji, Takafumi Ikeda, Hironori Haga, Toshiaki Saeki, Kenjiro Aogi, Tomoharu Sugie, Takayuki Ueno, Shinji Ohno, Hiroshi Ishiguro, Chizuko Kanbayashi, Takeshi Miyamoto, Yasuhiro Hagiwara, Masakazu Toi

**Affiliations:** 1 Department of Breast Surgery, Graduate School of Medicine, Kyoto University, Kyoto, Japan; 2 Department of Breast Surgery, Kyorin University School of Medicine, Mitaka, Japan; 3 Department of Breast and Endocrine Surgical Oncology, Tohoku University Graduate School of Medicine, Sendai, Japan; 4 Breast Oncology Center, Cancer Institute Hospital, Japanese Foundation for Cancer Research, Tokyo, Japan; 5 Department of Breast Oncology, Aichi Cancer Center Hospital, Nagoya, Japan; 6 Department of Breast and Endocrine Surgery, Nagoya University Graduate School of Medicine, Nagoya, Japan; 7 Department of Medical Oncology, National Cancer Center Hospital East, Kashiwa, Japan; 8 Department of Medical Oncology, Fukushima Medical University, Fukushima, Japan; 9 Department of Diagnostic Pathology, Kyoto University Hospital, Kyoto, Japan; 10 Breast Oncology Service, Saitama Medical University International Medical Center, Hidaka, Japan; 11 Department of Breast Oncology, National Hospital Organization Shikoku Cancer Center, Matsuyama, Japan; 12 Breast Surgery, Kansai Medical University Hospital, Hirakata, Japan; 13 Department of Breast Oncology, Niigata Cancer Center Hospital, Niigata, Japan; 14 Department of Breast Oncology, Gunma Prefectural Cancer Center, Ota, Japan; 15 Department of Biostatistics, Graduate School of Medicine, The University of Tokyo, Tokyo, Japan; 16 Tokyo Metropolitan Cancer and Infectious Disease Center, Komagome Hospital, 3-18-22, Honkomagome, Bunkyo-Ku, Tokyo, 113-8677 Japan

**Keywords:** Breast neoplasms, Chemotherapy, Adjuvant, Drug therapy, Receptors, Estrogen, Recurrence

## Abstract

**Purpose:**

The Phase III POTENT trial demonstrated the efficacy of adding S-1 to adjuvant endocrine therapy for estrogen receptor-positive, HER2-negative early breast cancer. We investigated the efficacy of S-1 across different recurrence risk subgroups.

**Methods:**

This was a post-hoc exploratory analysis of the POTENT trial. Patients in the endocrine-therapy-only arm were divided into three groups based on composite risk values calculated from multiple prognostic factors. The effects of S-1 were estimated using the Cox model in each risk group. The treatment effects of S-1 in patients meeting the eligibility criteria of the monarchE trial were also estimated.

**Results:**

A total of 1,897 patients were divided into three groups: group 1 (≤ lower quartile of the composite values) (*N* = 677), group 2 (interquartile range) (*N* = 767), and group 3 (> upper quartile) (*N* = 453). The addition of S-1 to endocrine therapy resulted in 49% (HR: 0.51, 95% CI: 0.33–0.78) and 29% (HR: 0.71, 95% CI 0.49–1.02) reductions in invasive disease-free survival (iDFS) events in groups 2 and 3, respectively. We could not identify any benefit from the addition of S-1 in group 1. The addition of S-1 showed an improvement in iDFS in patients with one to three positive nodes meeting the monarchE cohort 1 criteria (*N* = 290) (HR: 0.47, 95% CI: 0.29–0.74).

**Conclusions:**

The benefit of adding adjuvant S-1 was particularly marked in group 2. Further investigations are warranted to explore the optimal usage of adjuvant S-1.

**Supplementary Information:**

The online version contains supplementary material available at 10.1007/s10549-023-07099-4.

## Introduction

Randomized controlled trials have reported the survival benefit of endocrine therapy and chemotherapy in patients with estrogen receptor (ER)-positive, human epidermal growth factor receptor 2 (HER2)-negative breast cancer [1]. To practice precision medicine, it is essential to estimate the risk of disease recurrence and the benefit of treatment at the individual patient level. Prospective comparative trials have demonstrated that the recurrence score (RS) based on a 21-gene assay was useful for identifying patients with ER-positive, HER2-negative early breast cancer who could be spared multidrug cytotoxic chemotherapy [2–4]. Standard chemotherapy regimens for patients with primary breast cancer comprise anthracycline- and/or taxane-based chemotherapy [5–8]. Several clinical trials have investigated the efficacy of oral fluoropyrimidines, such as uracil-tegafur (UFT), as adjuvant chemotherapy in patients with breast cancer. A pooled analysis of six randomized trials revealed that the concurrent administration of 2-year UFT and tamoxifen improved the overall survival of patients with node-negative, ER-positive breast cancer [9]. In another pooled analysis of two randomized trials, UFT showed a similar therapeutic impact on recurrence-free survival and overall survival compared with classical cyclophosphamide, methotrexate, and fluorouracil (CMF) for early breast cancer patients with ER-positive and high-risk node-negative or node-positive disease [10–12]. In the United Kingdom TACT2 trial, where patients with node-positive or high-risk node-negative breast cancer were randomly assigned to accelerated or standard epirubicin followed by CMF or capecitabine [13], the capecitabine group showed a similar time to recurrence outcome compared with the CMF group. The CREATE-X trial demonstrated the potential survival benefit of adjuvant capecitabine among HER2-negative breast cancer patients with residual disease after preoperative systemic therapy (PST) [14]. The findings suggest that the addition of oral fluoropyrimidines to standard adjuvant endocrine therapy has the potential to improve survival outcomes in ER-positive, HER2-negative breast cancer.

The Phase III POTENT trial (jRCTs051180057/CRB5180002) investigated the efficacy of S-1 with adjuvant endocrine therapy in patients with stage I to IIIB ER-positive, HER2-negative early breast cancer [15]. S-1 is a triple combination drug comprising tegafur, gimeracil (a 5-fluorouracil inactivated enzyme inhibitor that is more potent than uracil), and oteracil potassium (an agent that reduces gastrointestinal toxicity). In the first-line setting of metastatic breast cancer treatment, S-1 monotherapy has been shown to be as effective as taxane [16]. The POTENT trial showed that one year of co-administration of S-1 with adjuvant endocrine therapy significantly improved 5-year invasive disease-free survival (iDFS) compared with adjuvant endocrine therapy alone (86.9% for S-1 arm vs. 81.6% for control arm, hazard ratio [HR]: 0.63, 95% confidence interval [CI]: 0.49–0.81) [15].

The monarchE trial underlined the importance of abemaciclib in the adjuvant setting of hormone receptor–positive, HER2-negative and node-positive breast cancer [17]. At a median follow-up of 42 months, patients who received abemaciclib plus endocrine therapy exhibited a better iDFS than those who received endocrine therapy alone (HR: 0.66, 95% CI: 0.58–0.76) [18, 19].

The POTENT trial included patients with a diverse range of risk of recurrence, excluding low risk of recurrence. There is some overlap in patient eligibility between the POTENT trial and the monarchE trial. We conducted an exploratory analysis investing the effect of S-1 by risk of recurrence, established by integrating conventional risk factors, to provide a better foundation for prescribing S-1.

## Material and methods

### Study design of the POTENT trial

This study is a post-hoc exploratory analysis of the POTENT trial. The POTENT trial was a multicenter, randomized, unblinded, controlled phase III trial in which patients were administered standard adjuvant endocrine therapy, either alone or with S-1 administered concurrently for one year [15]. Eligible patients were women aged 20–75 years with histologically diagnosed stage I to IIIB, ER-positive (≥ 1% by immunohistochemistry), HER2-negative (0 or 1 + by immunohistochemistry, or HER2/centromere enumeration probe ratio < 1.8 by fluorescence in situ hybridization) invasive breast cancer. Data on ER and HER2 expression, Ki-67, and histological grade were centrally reviewed by experienced pathologists at Kyoto University Hospital, Japan. Patients who underwent curative surgery were enrolled in the trial after the completion of either neoadjuvant/adjuvant chemotherapy and/or radiation therapy. Patients who received neoadjuvant endocrine therapy were also included in this trial. Patients with node-negative disease and protocol-defined low-risk features, or who were node-negative at disease presentation and who had achieved pathological complete response after neoadjuvant chemotherapy were excluded from this study.

All patients were treated with standard endocrine therapy. Patients in the S-1 group also received concurrent S-1 orally twice per day for 14 consecutive days followed by seven days off; this 21-day cycle was repeated for one year. The trial protocol was approved by the institutional review board of each study site. This study was conducted in accordance with the provisions of the Declaration of Helsinki and its later amendments. Written informed consent was obtained from all patients.

### Endpoint

The primary endpoint was iDFS, defined as the period from the treatment allocation date to either the confirmed recurrence date (excluding non-invasive cancer), date of confirmed development of other cancerous lesions, or date of death from any cause, whichever occurred first.

### Subgrouping

A continuous composite measure of recurrence risk (composite risk) for each patient was determined using a Cox proportional hazard model for iDFS incorporating age, tumor stage, nodal status, histological grade, ER positivity, and Ki-67, using data from the endocrine therapy only arm (*N* = 954) [20, 21]. After estimating the model parameters, the composite risk value was calculated for each patient by summing the parameter estimates corresponding to the patient’s observed clinicopathologic factor values. We divided the patients into three risk groups based on the distribution of the composite risk values in the control group: group 1, ≤ lower quartile; group 2, interquartile range; and group 3, > upper quartile.

We also investigated the impact of S-1 treatment on patients who met the eligibility criteria of the monarchE trial. For cohort 1, the eligibility criteria included patients with four or more positive nodes (cohort 1/N ≥ 4 +), or one to three positive nodes with at least one of the following: tumor size ≥ 5 cm or histological grade 3 (cohort 1/N1–3 +). For cohort 2, the eligibility criteria included patients with one to three positive nodes, Ki-67 ≥ 20%, and tumor size < 5 cm with Grade 1–2 (cohort 2).

### Statistical analysis

This was an exploratory analysis that was not pre-planned in the POTENT trial. The effects of S-1 treatment in each patient subgroup were estimated using the Kaplan–Meier method and a Cox proportional hazard model stratified by neoadjuvant/adjuvant chemotherapy use. Statistical analyses were conducted using JMP Pro (version 16.1.0; SAS Institute Inc., Cary, NC, USA) and Prism (version 9.5.1; GraphPad Software, LLC., San Diego, CA, USA).

## Results

**Fig. 1:**
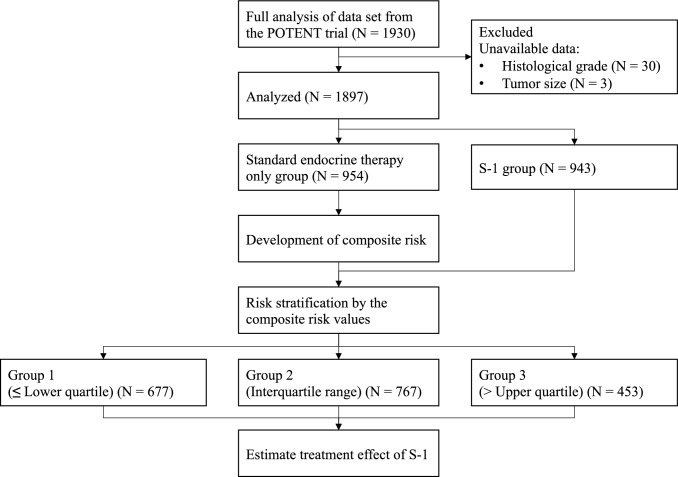
Flow diagram of patient selection. The full analysis cohort of the POTENT trial comprised 1,930 patients. Thirty-three patients were excluded from the analysis because of unavailable data (30 for histological grade and 3 for tumor stage); therefore, the final analysis included 1,897 patients. A continuous composite measure of recurrence risk (composite risk) was developed using the data from the control group (standard endocrine therapy only group). We divided the patients into three risk groups based on the distribution of the composite risk values in the control group: group 1, ≤ lower quartile; group 2, interquartile range; and group 3, > upper quartile. The treatment effects of S-1 in each patient group were estimated

The full analysis cohort of the POTENT trial comprised 1,930 patients. Thirty-three patients were excluded from the analysis because of unavailable data (30 for histological grade and 3 for tumor stage); therefore, the final analysis included 1,897 patients (Fig. 1, Table S1). One hundred and fifty-two patients in the endocrine therapy only arm and 100 patients in the S-1 arm experienced iDFS events during median follow-up time of 52.2 months.

**Fig. 2:**
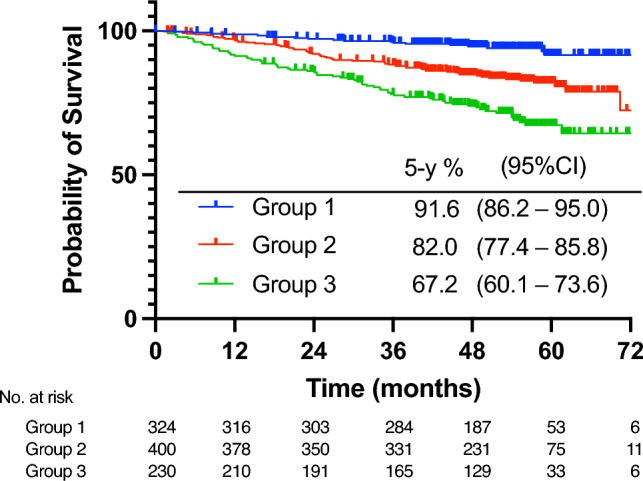
Kaplan–Meier estimate of invasive disease-free survival in the endocrine therapy only group according to the patient group defined by the composite risk value. Group 1, ≤ lower quartile of the composite risk value; Group 2, interquartile range; Group 3, > upper quartile. CI, confidence interval; iDFS, invasive disease-free survival; 5-y, 5-year

Table 1 presents the multivariate Cox regression model for composite risk using data from the endocrine therapy only arm. Age category was excluded from the multivariate model because it did not add a significant prognostic value to the model. The distribution of the composite risk values in the endocrine therapy only arm is shown in Figure S1 (median, 1.76; interquartile range, 1.35–1.93). The composite risk values as continuous values correlated with iDFS (HR: 2.72, 95% CI 2.11–3.49). We divided the endocrine therapy only arm into three groups based on the composite risk values. The 5-year iDFS rates in groups 1, 2, and 3 were 91.6%, 82.0%, and 67.2%, respectively (Fig. 2). We employed the same cut-off value for composite risk to the S-1 arm. The clinicopathological characteristics of the patients in the three groups are summarized in Table 2. The patient characteristics were well balanced between the treatment arms in each composite risk group. The proportion of patients who received neoadjuvant/adjuvant chemotherapy in groups 1, 2, and 3 were 40%, 59%, and 73%, respectively. Patients who received neoadjuvant or adjuvant chemotherapy had a significantly worse prognosis than those who did not receive such chemotherapy (5-year iDFS rate 77.7% vs. 86.7%, *P* < 0.01). The treatment effect of S-1 was evaluated by adjusting the chemotherapy use in the Cox proportional hazard model. Two-thirds of patients in group 1 had cT1 disease and about half had lymph node metastasis. Most patients in group 1 had breast cancer of histological grade 1–2 (99%) and Ki-67 < 14% (91%). Seventy-two percent of patients classified in group 2 presented with cT2 disease and 64% exhibited nodal metastasis. Most patients had breast cancer of grade 2–3 (98%) and Ki-67 < 30% (92%). Eighty-nine percent of patients with cT3–4 disease were classified into group 3, and 77% of patients in group 3 had nodal metastasis. More patients with grade 3 or Ki-67 ≥ 30% were classified into group 3 compared to other groups.

**Table 1:** Cox regression for composite risk using the endocrine therapy only group

Factors	Parameter estimate	SE	*χ*^2^	HR	95%CI
Clinical tumor stage					
cT1	0 (ref)				
cT2	0.42	0.19	4.9	1.5	(1.0–2.2)
cT3-4	1.16	0.26	18.5	3.2	(1.9–5.3)
Nodal metastasis					
Yes	0.49	0.18	7.4	1.6	(1.1–2.3)
No	0 (ref)				
ER-positivity					
1–9%	1.00	0.48	3.4	2.7	(1.1–6.9)
≥ 10%	0 (ref)				
Histological grade					
1	0 (ref)				
2	0.86	0.43	5.1	2.4	(1.0–5.5)
3	1.34	0.49	9.1	3.8	(1.5–10.0)
Ki-67					
< 14%	0 (ref)				
≥ 14%, < 30%	0.17	0.24	0.5	1.2	(0.7–1.9)
≥ 30%	0.55	0.32	2.9	1.7	(0.9–3.2)

*ER* estrogen receptor, *HR* hazard ratio, *CI* confidence interval

**Table 2:** Distribution of clinicopathological factors between patients groups defined by composite risk value

Factors		Group 1 (*N* = 677)				Group 2 (*N* = 767)				Group 3 (*N* = 453)			
		Control (*N* = 324)		S-1 (*N* = 353)		Control (*N* = 400)		S-1 (*N* = 367)		Control (*N* = 230)		S-1 (*N* = 223)	
		*N* (%)	*N* (%)	*N* (%)	*N* (%)	*N* (%)	*N* (%)	*N* (%)	*N* (%)	*N* (%)	*N* (%)	*N* (%)	*N* (%)
Age	Median (range)	53	(29–75)	53	(29–75)	50	(30–75)	52	(29–75)	51	(27–75)	51	(29–74)
Menopause													
Pre		147	45%	155	44%	212	53%	183	50%	113	49%	113	51%
Post		177	55%	198	56%	188	47%	184	50%	117	51%	110	49%
Tumor size													
cT1		207	64%	240	68%	107	27%	92	25%	35	15%	38	17%
cT2		114	35%	113	32%	286	72%	266	73%	114	50%	106	48%
cT3-4		3	1%	0	0%	7	2%	9	3%	81	35%	79	35%
Nodal metastasis													
Yes		169	52%	190	54%	260	65%	233	64%	171	74%	176	79%
No		155	48%	163	46%	140	35%	134	37%	59	26%	47	21%
ER													
1–9%		0	0%	0	0%	0	0%	1	0%	11	5%	9	4%
≥ 10%		324	100%	353	100%	400	100%	366	100%	219	95%	214	96%
Chemotherapy													
Yes		126	39%	144	41%	239	60%	212	58%	163	71%	166	74%
No		198	61%	209	59%	161	40%	155	42%	67	29%	57	26%
Grade													
1		99	31%	97	28%	7	2%	10	3%	0	0%	0	0%
2		222	69%	255	72%	292	73%	270	74%	71	31%	62	28%
3		3	1%	1	0%	101	25%	87	24%	159	69%	161	72%
Ki67													
< 14%		292	90%	323	92%	176	44%	157	43%	54	24%	44	20%
≥ 14%, < 30%		32	10%	30	9%	189	47%	183	50%	83	36%	86	39%
≥ 30%		0	0%	0	0%	35	9%	27	7%	93	40%	93	42%

*ER* estrogen receptor

**Fig. 3:**
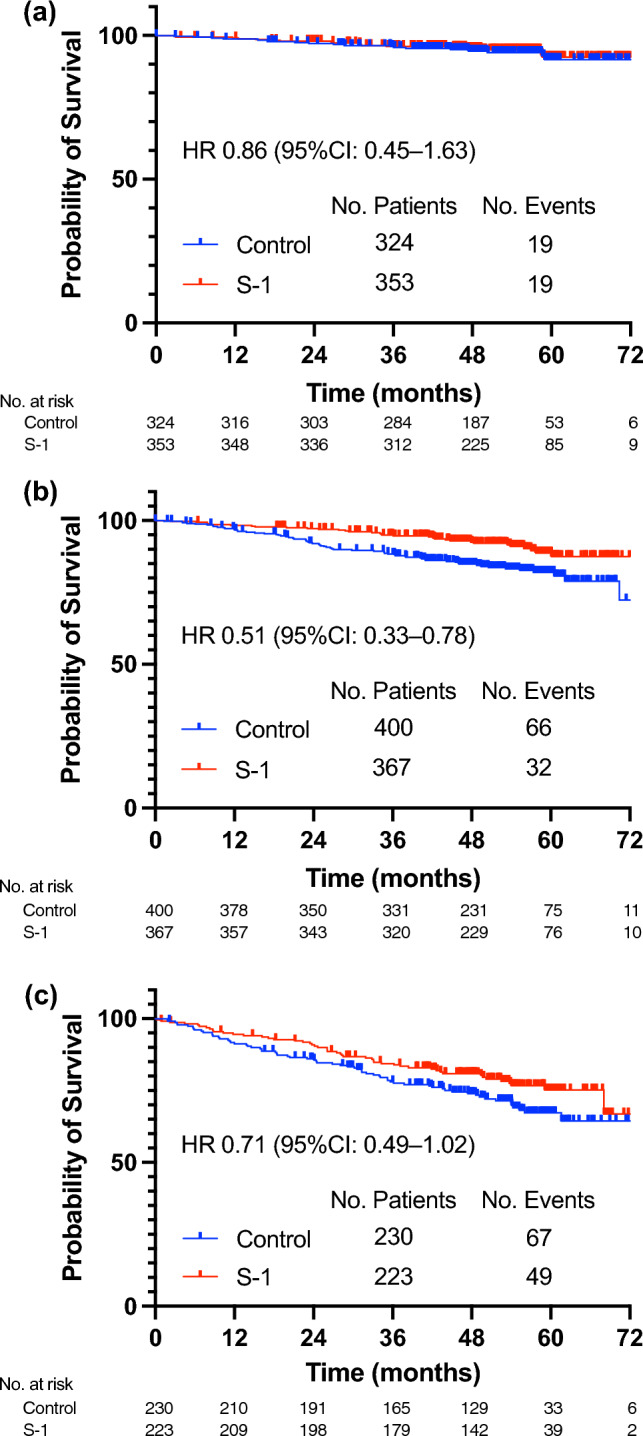
Effects of S-1 treatment in each risk group. Kaplan–Meier estimate of invasive disease-free survival. **A** Treatment effect of S-1 in the Group 1, **B** Treatment effect of S-1 in the Group 2, **C** Treatment effect of S-1 in the Group 3. *HR* hazard ratio, *CI* confidence interval, *iDFS* invasive disease-free survival, *5-y* 5-year

We proceeded to estimate the treatment effect of S-1 in each composite risk group. The effects of adding S-1 on endocrine therapy varied by the risk group. There was no significant benefit from S-1 in group 1 (HR: 0.86, 95% CI 0.45–1.63), as both the control and S-1 arms demonstrated excellent 5-year iDFS rates of 91.6% (95% CI: 86.2–95.0%) and 92.5% (95% CI: 87.9–95.2%), respectively (Fig. 3A). The addition of S-1 to adjuvant endocrine therapy resulted in a remarkable improvement in iDFS in group 2 (Fig. 3B). The HR of the effects of S-1 in group 2 was 0.51 (95% CI 0.33–0.78). In group 3, there was also an improvement in iDFS with the addition of S-1 (HR: 0.71, 95% CI 0.49–1.02), although the effect of S-1 was smaller than that in group 2 (Fig. 3C). In the control and S-1 arms of group 2, the 5-year iDFS rates were 82.0% (95% CI: 77.4–85.8%) and 88.7% (95% CI: 83.9–92.2%) respectively. In group 3, the corresponding rates were 67.2% (95% CI: 60.1–73.6%) and 75.3% (95% CI: 68.2–81.2%), respectively.

**Fig. 4:**
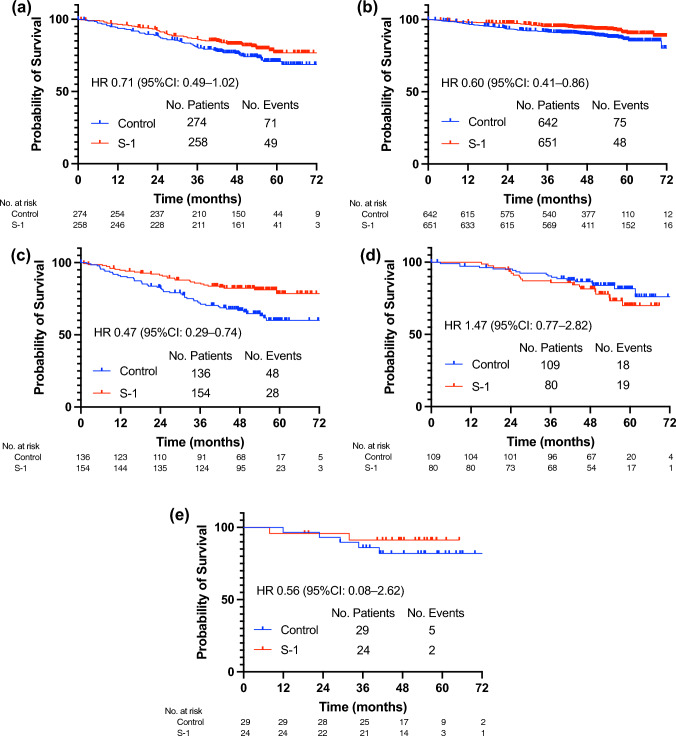
Effects of S-1 treatment in patients fulfilling monarchE criteria. Kaplan–Meier estimate of invasive disease-free survival. **A** patients who met monarchE criteria, **B** patients who did not meet monarchE criteria, **C** patients who met monarchE cohort 1 criteria with one to three positive nodes, **D** patients who met monarchE cohort 1 criteria with four or more positive nodes, **E** patients who met monarchE cohort 2 criteria

A total of 532 patients met the eligibility criteria for the monarchE trial. The proportion of patients meeting the monarchE criteria for groups 1, 2, and 3 were 7%, 20%, and 74%, respectively (Table S2). The addition of S-1 to endocrine therapy led to a modest improvement in iDFS in patients who met the monarchE criteria (Fig. 4A). The HR of the effects of S-1 in the monarchE subgroup was 0.71 (95% CI 0.49–1.02). The addition of S-1 to endocrine therapy resulted in 40% reduction in iDFS events in patients who did not meet the monarchE criteria (HR of 0.60, 95% CI 0.41–0.86) (Fig. 4B). The monarchE subgroup was further subdivided into cohort 1/N1–3 +, cohort 1/N ≥ 4 + , and cohort 2, and the treatment effect of S-1 was assessed in each subgroup (Fig. 4C–E). Adjuvant treatment with S-1 resulted in a 53% reduction in iDFS events in the cohort 1/N1–3 + group (HR of 0.47, 95% CI 0.29–0.74). However, treatment with S-1 had no observable effect in the cohort 1/N ≥ 4 + subgroup or in cohort 2.

## Discussion

The present investigation has revealed that patients in group 2, as defined by integration of the clinicopathological factors of patient data from the POTENT trial, experienced a major therapeutic benefit from the addition of S-1 to adjuvant endocrine therapy. An additional analysis indicated that the incorporation of S-1 has resulted in an improvement in iDFS among patients meeting the monarchE cohort 1 criteria without extensive nodal involvement.

In this study, we integrated the conventional prognostic factors, including tumor size, nodal status, ER positivity, histological grade, and Ki-67, and developed a composite risk value to estimate patient prognosis. We divided the patients into three risk groups based on quartiles of the composite risk values. However, patient numbers in group 1 and 3 were different and the numbers in group 1 and 2 were close. Composite risk values are score values calculated from a limited number of clinicopathological factors, so they are in fact ordinal rather than continuous. As shown in Figure S1, there were a reasonable number of patients with similar composite risk values, especially around the interquartile range. Therefore, the distribution of the number of patients changes significantly depending on which group the quartile is included in. Although the clinical validity of Ki-67 remains uncertain, Ki-67 values are often considered when deciding on suitable adjuvant chemotherapy for patients with ER-positive, HER2-negative breast cancer [22–24]. In our study, Ki-67 was obtained from central pathology assessment to avoid inter-laboratory variability. Although young age has been regarded as a prognostic factor in patients with ER-positive breast cancer [25, 26], age was not associated with iDFS in the current study. Younger patients or pre-menopausal patients in the POTENT trial were actually administered neoadjuvant or adjuvant chemotherapy more frequently. The higher proportion of patients who were treated with chemotherapy may explain why young age did not appear to have prognostic significance in the current study. However, neither age nor pre-menopausal status appear to have prognostic significance in the multivariate analysis incorporating neoadjuvant/adjuvant chemotherapy administration (data not shown).

Our composite risk model identified a subgroup of patients with a favorable prognosis, even though the POTENT trial did not actually include patients at low risk of recurrence, such as low-grade stage I disease. Patients classified in group 1 had an iDFS rate of 91.6% for the control arm and 92.5% for the S-1 arm at 5 years. In this group with a particularly favorable prognosis, iDFS events were too infrequent to detect any advantages of adding S-1 to adjuvant endocrine therapy. Most patients in group 1 had breast cancer of histological grade 1–2 (99%) and Ki-67 < 14% (91%). In the TAILORx trial, node-negative patients with RS < 26 did not derive a survival benefit from multidrug adjuvant chemotherapy [3]. Additionally, the Rx-PONDER trial demonstrated that postmenopausal patients with 1–3 positive nodes and RS < 26 did not benefit from adjuvant chemotherapy either [2]. In the MINDACT trial, 48% of clinically high-risk and genomically low-risk patients had node-positive disease, but the 5-year rate of distant disease-free survival of these patients was 95.1% (95% CI: 93.1–96.6%) even in the absence of multidrug adjuvant chemotherapy [27]. For this patient subgroup with low-grade or low-proliferative disease, detecting any benefit from the addition of S-1 to adjuvant endocrine therapy is challenging.

The impact of the treatment effect of S-1 was more pronounced in group 2 compared to group 3. The majority of patients classified in group 2 presented with cT1–T2 disease and nearly two-thirds exhibited nodal metastasis. Most patients in group 2 had grade 2–3 breast cancer and a Ki-67 labeling index < 30%. Group 3 included more patients with advanced stage, high grade, and high proliferative disease compared to group 2. In general, patients with a higher grade or higher proliferative index showed a greater benefit from chemotherapy. The different modes of action of S-1 and other chemotherapy agents may explain this discrepancy. In the aforementioned pooled analysis of adjuvant UFT trials, patients with cT1–2, node-negative, and ER-positive breast cancer showed a greater improvement in overall survival with the combination of UFT and tamoxifen compared with tamoxifen alone (HR 0.28 vs. 0.44) [9]. In the pooled analysis of the N-SAS BC01 trial and the CUBC trial, in patients with ER-positive early breast cancer, UFT was shown to be non-inferior to CMF in terms of recurrence-free survival and overall survival [10]. The NSAS BC01 trial included patients with node-negative and grade 2–3 disease, and 95% of patients had cT1–T2 disease. The CUBC trial included patients with node-positive disease, and 85% of patients had cT1–T2 disease and 72% had one to three positive nodes. Since the clinicopathological characteristics of patients in group 2 shared many similarities with those included in previous clinical trials demonstrating the efficacy of UFT, it seems reasonable to impute that S-1 had a significant effect in group 2. In addition, since the POTENT trial investigated the effect of adding S-1 on endocrine therapy, this study does not estimate the effect of S-1 in itself, excluding the effects of endocrine therapy. The interaction between the composite risk values as continuous values and S-1 treatment was not significant (data not shown). This means that the effect of adding S-1 on endocrine therapy does not increase as the composite risk values increase and is consistent with our finding that the effect of S-1 was more pronounced in group 2 than in group 3.

The treatment effect of S-1 was modest in patients meeting the monarchE criteria. In this patient cohort, an improvement in iDFS by the addition of S-1 was observed in cohort 1/N1–3 + , but not in cohort 1/N ≥ 4 + . Although these results are based on a limited number of cases and events and should be interpreted with caution, S-1 may not have a sufficient therapeutic effect in patients with extensive nodal metastasis. The CREATE-X trial targeted more advanced breast cancer than the POTENT trial, and there was no benefit from the additional administration of capecitabine after preoperative chemotherapy in the patient subgroup with ER-positive breast cancer. The efficacy of oral fluoropyrimidine in patients with advanced lymph node metastasis warrants further investigation.

The current study was conducted using data from a randomized controlled trial. We utilized 98% of the full analysis data set of the POTENT trial, and the clinicopathological characteristics of the S-1 and control arms were well balanced in each risk group. This enabled us to evaluate the effects of S-1 treatment with minimal selection bias. A limitation of this study was that it was a subgroup analysis with a limited number of events. Therefore, the results of this study should be interpreted with caution. The POTENT trial was terminated at the time of the interim analysis because the primary endpoint was met. In addition, the POTENT trial included a large number of patients at intermediate risk of recurrence, which limited the number of events obtained. Due to the overlapping targeted populations in the POTENT and monarchE trials, the question as to whether S-1 or abemaciclib should be administered as adjuvant therapy frequently arises in clinical practice. Thus, it is clinically significant to assess the therapeutic impact of S-1 based on the risk of recurrence. Our composite risk value has not been validated its prognostic value in the other clinical dataset, and should only be used to estimate the effect of adding S-1 on endocrine therapy in the POTENT trial.

Our composite risk value effectively categorized the patients who participated in the POTENT trial into three distinct risk groups. The therapeutic impact of incorporating S-1 into adjuvant endocrine therapy may vary depending on the risk of recurrence, and notable results were achieved in group 2. An additional analysis showed that patients without extensive nodal involvement who met the criteria of monarchE cohort 1 may benefit from the addition of S-1 to adjuvant endocrine therapy. Further investigations are warranted to explore the optimal usage of adjuvant oral fluoropyrimidines.

### Supplementary Information

Below is the link to the electronic supplementary material.

Supplementary file1 (DOCX 44 kb)

## Data Availability

The data on which this article is based will be made available if a reasonable request is made to the corresponding author. Requests for data access should be made in writing and be addressed to the corresponding author, including details of how the data will be used. Sharing of data will be considered based on scientific merit, feasibility, and timeliness of the request.

## Abbreviations

CI: Confidence interval
CMF: Cyclophosphamide, methotrexate, and fluorouracil
ER: Estrogen receptor
HER2: Human epidermal growth factor 2
HR: Hazard ratio
iDFS: Invasive disease-free survival
Ki-67: Ki-67 labelling index
PGV: Pathogenic germline variant
PST: Preoperative systemic therapy
RS: Recurrence score
UFT: Uracil-tegafur
