# Supplementary material for: A risk-based subgroup analysis of the effect of adjuvant S-1 in estrogen receptor-positive, HER2-negative early breast cancer
Source: Breast Cancer Res Treat. 2023 Sep 7;202(3):485–96. doi: 10.1007/s10549-023-07099-4 (PMC10564670; doi:10.1007/s10549-023-07099-4)
Supplement: Supplementary file 1 — Supplementary file1 (DOCX 44 kb) [file 10549_2023_7099_MOESM1_ESM.docx]

**List of Supporting Information**:

Tables:

Table S1: Patient characteristics of the POTENT trial

Table S2: Proportion of patients who met the monarchE criteria in the risk groups

Figures:

Figure S1. Frequency distribution of the composite risk vales in the endocrine therapy only arm

**Table S1: Patient characteristics of the POTENT trial**

| **Factors** |  | **Endocrine therapy only group (N=954)** |  | **Endocrine therapy plus S-1 group (N=943)** |  |  |
| --- | --- | --- | --- | --- | --- | --- |
|  |  | **N** | **%** | **N** | **%** | **P-value** |
| **Age** | **median (range)** | 51 | (27–75) | 52 | (29–75) | 0.87 |
| **Clinical tumor stage** | **cT1** | 349 | 37% | 370 | 39% | 0.49 |
|  | **cT2** | 514 | 54% | 485 | 51% |  |
|  | **cT3-4** | 91 | 10% | 88 | 9% |  |
| **Nodal metastasis** | **Yes** | 600 | 63% | 599 | 64% | 0.78 |
|  | **No** | 354 | 37% | 344 | 36% |  |
| **ER-positivity** | **1-9%** | 11 | 1% | 10 | 1% | 0.85 |
|  | **≥ 10%** | 943 | 99% | 933 | 99% |  |
| **Neo/Adjuvant chemotherapy** | **Yes** | 528 | 55% | 522 | 55% | 1.00 |
|  | **No** | 426 | 45% | 421 | 45% |  |
| **Histological grade** | **1** | 106 | 11% | 107 | 11% | 0.85 |
|  | **2** | 585 | 61% | 587 | 62% |  |
|  | **3** | 263 | 28% | 249 | 26% |  |
| **Ki-67** | **< 14%** | 522 | 55% | 524 | 56% | 0.89 |
|  | **≥ 14%, < 30%** | 304 | 32% | 299 | 32% |  |
|  | **≥ 30%** | 128 | 13% | 120 | 13% |  |

**Table S2: Proportion of patients who met the monarchE criteria in the risk groups**

|  | **Group 1** |  | **Group 2** |  | **Group 3** |  |
| --- | --- | --- | --- | --- | --- | --- |
|  | **N** | **%** | **N** | **%** | **N** | **%** |
| **non-monarchE** | 600 | 89% | 584 | 76% | 109 | 24% |
| **monarchE** | 45 | 7% | 153 | 20% | 334 | 74% |
| **Unclassified** | 32 | 5% | 30 | 4% | 10 | 2% |

**Figure S1. Frequency distribution of the composite risk vales in the endocrine therapy only arm**
